# Developing a Threshold Concept Assessment Rubric: Using the Johnstone’s Triangle Framework for Understanding Intermolecular Forces

**DOI:** 10.1021/acs.jchemed.4c00236

**Published:** 2024-10-02

**Authors:** Simbarashe Nkomo, Alia Bly

**Affiliations:** Division of Natural Science and Mathematics, Oxford College of Emory University, Oxford, Georgia 30054, United States

**Keywords:** Threshold Concept, Johnstone’s Triangle, Intermolecular Forces, Assessment Rubric, First/Second-Year Undergraduates, Backward Course Design

## Abstract

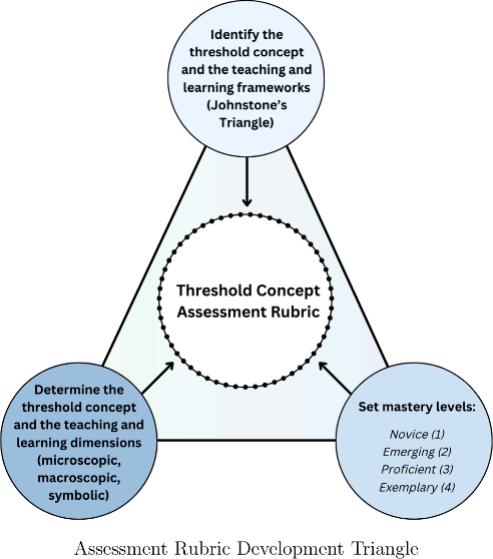

In undergraduate science education, laboratory courses stand as essential cornerstones of experiential learning. Chemistry laboratory courses offer students unique hands-on experiences that bridge the gap between theoretical knowledge and practical application. The journey through the undergraduate chemistry curriculum is paved with a series of conceptual gateways known as threshold concepts that can dramatically shape a student’s understanding and success. We identified the idea of intermolecular forces (IMFs) as a threshold concept to students’ ability to link molecular structures, properties, and applications to real-world problems such as extraction and separation of compounds. The development of course-specific pedagogical tools can provide students with the scaffolding necessary for the transition from novice to expert-level disciplinary comprehension. This work presents the development process of a Threshold Concept Assessment Rubric (TCAR) based on Johnstone’s triangle framework and discusses its application for evaluating students’ progress in overcoming a threshold concept. The rubric is used in a 200-level multilayer laboratory course that is intentionally designed with intermolecular forces as the central theme. We analyze the role and structure of different questions to provide a holistic assessment of students’ learning processes using sample assignments. Furthermore, we demonstrate how insights from statistical analyses can highlight areas in which students struggle to gain expert or exemplary-level understanding of IMFs. This rubric development approach can be applied to other threshold concepts.

## Introduction

Chemistry education at the undergraduate level seeks to conceptualize theoretical phenomena at scales ascertainable to students developing their perceptions of the interconnections of molecular interactions. For emerging chemistry students, success is largely defined by overcoming threshold concepts, which provide intellectual frameworks for more multidimensional expressions of chemical phenomena.^1,2^ Threshold concepts have been identified as bridging gaps between theory and practice.^3^ Examples identified by some scholars include chemical bonding,^4^ mole concept, atomic structure,^5^ redox reactions,^6^ and free energy.^7^ The identification of concepts varies due to a diversity of factors that impact the learning process. Among these factors are pedagogical methods, resources for supporting concept formation, and education level.^8^ We identified the application of intermolecular forces (IMFs) to be a threshold concept for first- and second-year undergraduate thinkers. Application of intermolecular forces requires students to engage with chemical structures, molecular properties, and applications on conceptual and observable levels.^7^ Intermolecular forces are the bridge connecting physical and chemical properties to molecular structure. The various levels at which IMFs can be examined are well-illustrated by Johnstone’s triangle.^9^

Johnstone’s triangle, devised in the 1980s, provides a framework for student comprehension of IMFs through establishing clear dimensions by which to perceive molecular characteristics.^9−12^ Effective interpretation of different representations within a given problem can lead to improved comprehension when multiple facets of a question must be considered simultaneously.^9,13,14^ However, without a preemptive understanding of how best to approach the multilevel nature of IMFs, students may struggle to conceptualize chemical interactions and applications at both micro and macro scales.^13,15^ Chemistry as a subject is considerate of not only semiotics but also the language and methodology used to express such representations, and Johnstone’s triangle attempts to unify submicroscopic, macroscopic, and symbolic levels to build a cohesive expression of chemical phenomena.^10,16^

**Figure 1:**
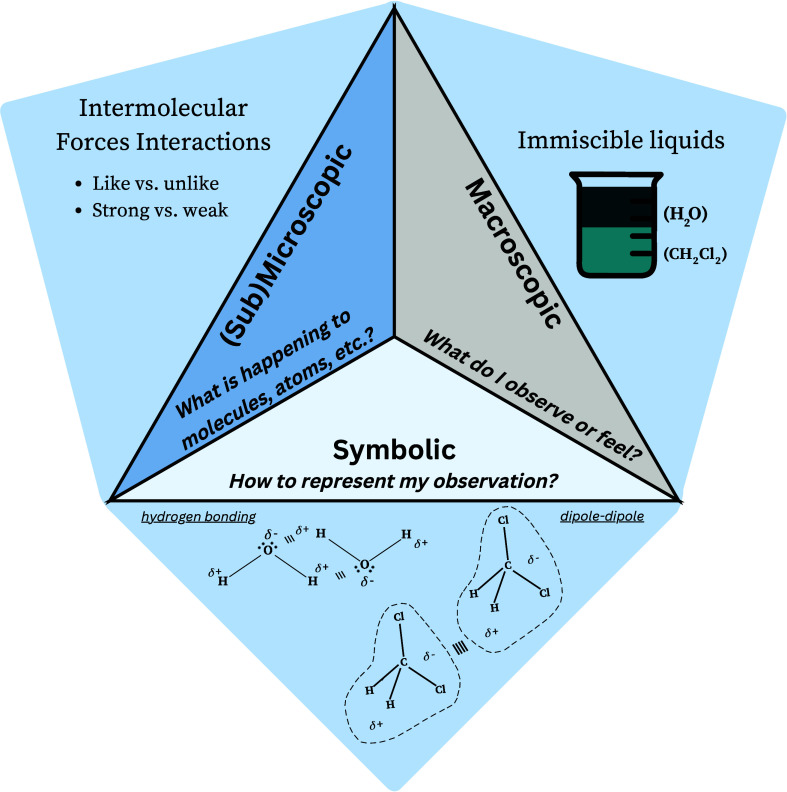
Dimensions of Johnstone’s triangle and examples of application to IMFs.

The American Association of Colleges and Universities’ Valid Assessment of Learning in Undergraduate Education (VALUE) rubrics focus on holistic assessment of soft skills in multiple disciplines. These rubrics provide a basis to creating assessment tools geared toward classroom-specific expectations.^17,18^ Various rubrics have been developed and implemented in chemistry to assess skills such as problem-solving,^19^ processing (critical thinking and information processing),^20^ hands-on laboratory skills,^21^ and metacognitive reflection.^22^ In these studies, the different dimensions required to master the skills are used as dimensions of the rubric. We identified the Johnstone’s triangle framework as a basis for the development of a similarly holistic rubric. As illustrated in Figure 1, the framework breaks core concepts into three dimensions. A student’s mastery level corresponds to their ability to seamlessly navigate through the three dimensions of the triangle.

This study presents the development of a Threshold Concept Assessment Rubric (TCAR) based on Johnstone’s triangle for assessing students’ progress toward overcoming the IMF threshold concept in a 200-level chemistry undergraduate laboratory course. Designing scoring rubrics attuned to the characteristics of a given threshold concept allows educators to not only assess their students but also to better convey their expectations for content mastery within pre-existing subject area frameworks.^23^

### Context

Oxford College is a division of Emory University that provides a small liberal arts college experience in the first two years of an Emory University education. The small classes foster collaboration among faculty and students. In Fall 2017, Emory introduced the “Chemistry Unbound” curriculum, which reorganized the foundational courses into themes rather than the traditional compartmentalization of chemistry: general, analytical, physical, inorganic, and organic. Instead, the curriculum focuses on connecting concepts across these areas of chemistry. This curriculum change process created opportunities for redesigning the laboratory curriculum, as well.

**Figure 2:**
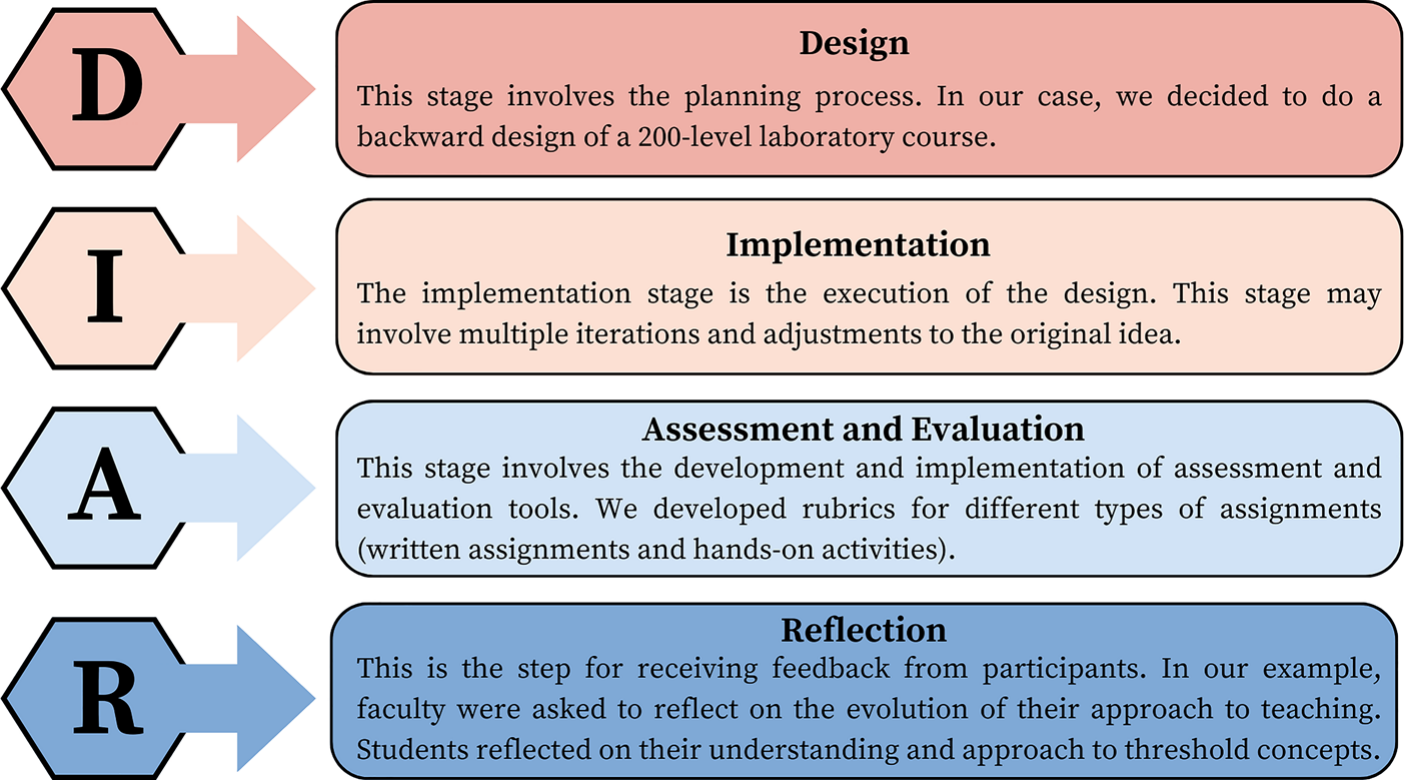
Curriculum innovation process.

The main stages of curriculum innovation are design, implementation, and evaluation. During the design stage, the chemistry faculty decided to engage in a backward design process^24^ of a 200-level chemistry laboratory course centered on helping students overcome a threshold concept that connects the first three courses in the curriculum sequence. Through analyses of students’ performance, feedback, and reflections, instructors identified IMFs as a threshold concept for the culminating lab experience design. Understanding IMFs is fundamental in chemistry. The course (Chem 202L) includes topics such as solubility, liquid–liquid extraction, and chromatography, which require an understanding of IMFs. The implementation process involved a series of iterations and adjustments to developed assignments and pedagogical approaches. We developed a range of assessment tools including pre- and postlab assignments, interview questions, and lab practical skills. This work focuses on the evaluation process—particularly the rubric development of a rubric for assessing students’ learning progress. The details of the design stage are found in the referenced publication,^25^ and the implementation and reflection stages will be discussed in our future work. Figure 2 summarizes the curriculum innovation process with some references to our example.

## Rubric Development Process

In this section, we discuss our rubric development process after identifying IMFs as the focus threshold concept and the Johnstone’s triangle as the framework for progress assessment. The stages are discussed in detail in the subsections below in the following order: assignment development, scoring rubric development, rubric norming, sample statistical analysis, and rubric generalization.

### Assignment Development

**Figure 3:**
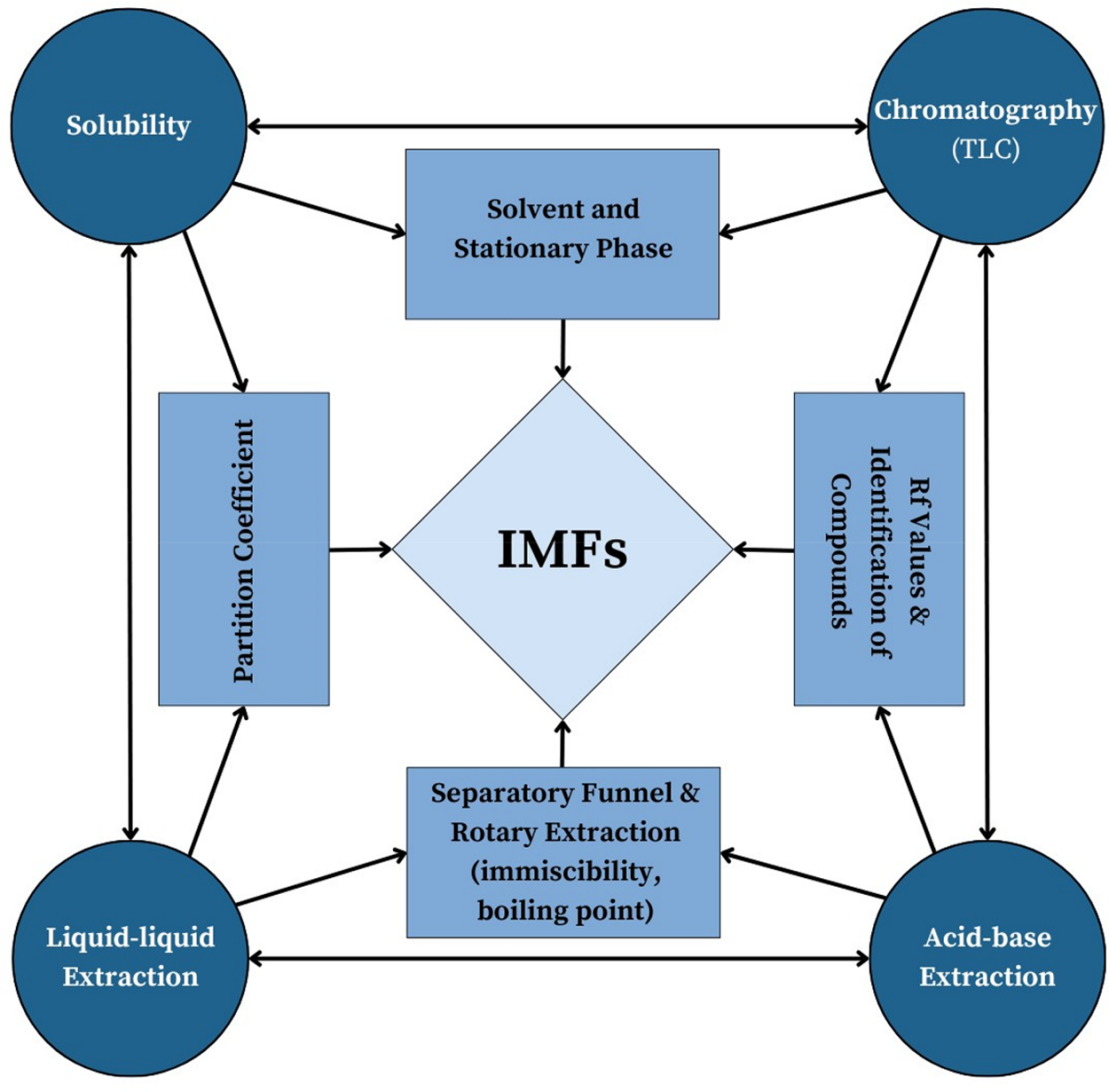
Examples of concepts in chemistry and associated laboratory activities connected to IMFs.

The laboratory course Chemistry 202—*Principles of Reactivity*—examines IMFs as a basis for a fundamental understanding of concepts in chemistry. The course connects IMFs to real-world applications through experiments on solubility, partition coefficients, solute extraction methods, Thin Layer Chromatography (TLC), and other key concepts. For example, the octanol–water partition coefficient (Kow) is used in drug toxicity studies, solute extraction methods are used to extract synthesized drugs from solvents, and TLC is used to identify compounds in mixtures.^26−28^ The concept map in Figure 3 demonstrates how IMFs underpin these activities. Accordingly, we identified IMFs to be a threshold concept in the context of our lab curriculum design.

Aligning the learning framework was central to our assignment development, and we were mindful that one assignment would not do everything. We intentionally planned to provide multiple ways and opportunities for students to demonstrate their understanding of IMFs. To further explore the concepts in Figure 3, we designed lab quizzes, workshop activities, interviews, lab exams, and practical lab exams. In this section, we use the pre- and postlab assessment as an example to illustrate an assignment development process.

Prior to the starting the in-person laboratory activities at the beginning of the semester, students complete a preassessment quiz consisting of six questions. Each question examines IMFs within the range of the three dimensions of Johnstone’s triangle. Questions range from application-based to the explicit identification of IMFs present within a given pair of molecules, thus allowing students to demonstrate their understanding of chemical phenomena at multiple scales.

**Figure 4:**
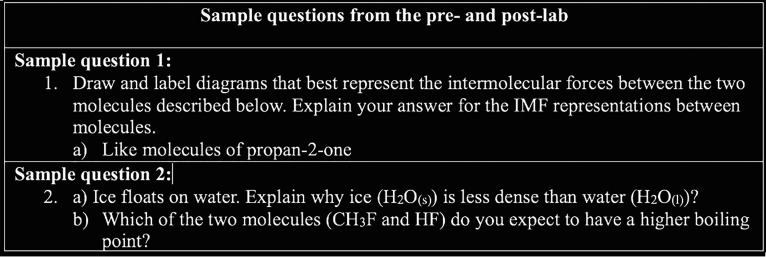
Examples of questions included on the pre- and post-assessments prompting (sub)microscopic, symbolic, and macroscopic analysis.

This prelab quiz serves as a basis to evaluate student understanding of IMFs upon entry to the course. Students are also expected to participate in a postexamination composed of the same six questions and a new question related to their laboratory practical exam. With these examinations, we generated relevant benchmarks for student thinking process and understanding of IMFs. Figure 4 shows examples of questions in the lab quiz, which connect the microscopic and symbolic dimensions of Johnstone’s triangle. Sample question 1 asks students to represent their understanding of what is happening at the microscopic level and also to explain their representation, which is the symbolic dimension. In sample question 2, students are asked about the connection with the third dimension, which is the observable. Coming into this lab, many students have seen and know that ice is less dense than liquid water, but they have not all considered explaining this phenomenon using IMFs. Providing an explanation requires students to consider what is happening at the microscopic level and how to communicate their representation of microscopic interactions (symbolic) that lead to the observed phenomenon (macroscopic level). Part B of the question also focuses on the same connections as the observable, but it is phrased in a predictive way to prompt a different navigation path around the triangle. Additional questions to provide students with opportunities to demonstrate their navigation of the triangle’s three dimensions are included in Section SIA of the Supporting Information. As stated earlier, we developed multiple assignments to provide opportunities for different modes of expressing mastery of the IMFs.

### Setting Mastery Levels (Co-dimensions)

**Figure 5:**
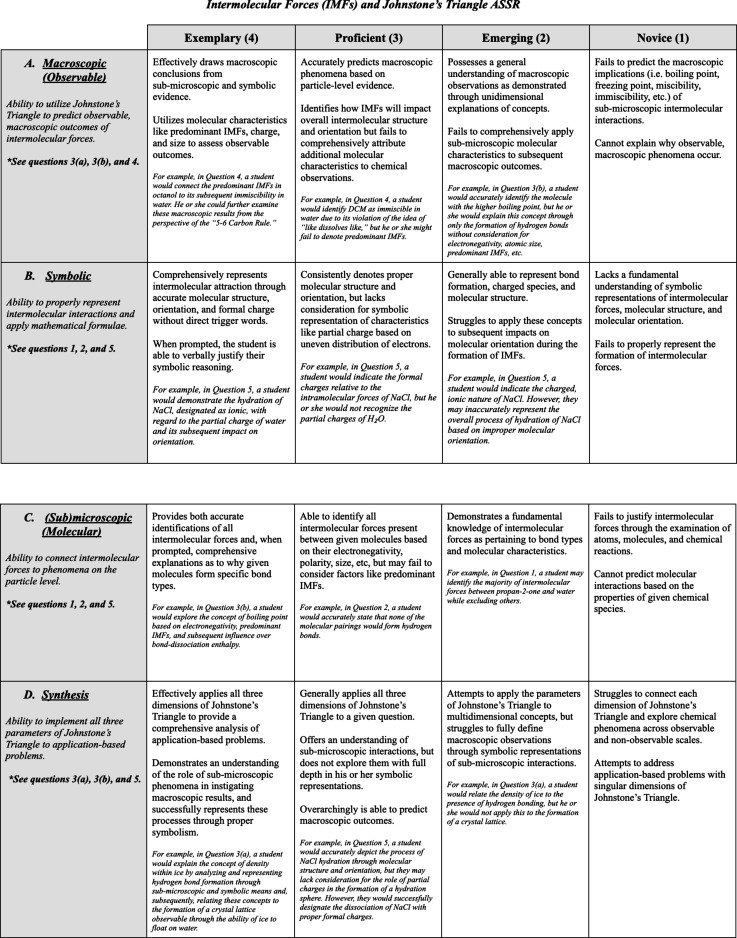
Developed Assignment Specific Scoring Rubric for the pre- and postlab assessments.

We generally expected the students’ ability to navigate through the elements of Johnstone’s triangle to improve with increasing exposure to the teaching and learning framework. For both feedback to the students and evaluation of our goal to help them cross the threshold barrier, we categorized the learning progress levels as novice, emerging, competent, and exemplary. We identify these mastery levels as codimensions, as they indicate a level within a dimension of the rubric. Exemplary level is where one seamlessly navigates through the dimensions of Johnstone’s triangle. The other categories are defined relative to this final level. It is important to note that the descriptions of these mastery levels may vary depending on the threshold concept. Our description of the levels is shown in Figure 5. Setting mastery levels completes the components of the rubric development triangle in the abstract.

### Developing an Assignment Specific Scoring Rubric (ASSR)

Holistic assessment of student knowledge as demonstrated by pre- and postexaminations was first conducted on a question-by-question basis to determine learning objectives in relation to Johnstone’s triangle. During this phase, a draft rubric was created to capture the different levels of questioning and the alignment of the questions with the dimensions of Johnstone’s triangle (Figure 5).

Analyzing how the pre- and postlab quiz questions related to the learning objectives led to an assessment rubric with four dimensions: *macroscopic* (or *observable*), *microscopic* (or *molecular*), *symbolic*, and *synthesis*. The terms macroscopic and microscopic are conveniently used to illustrate the scale shift. Each dimension was assigned a letter label ranging from A to D (Figure 5). The questions from the quiz (Supporting Information Section SIA) were specifically assigned to one or more dimensions. For example, a question in which students were prompted to predict miscibility of two compounds was used as an indicator of microscopic and macroscopic comprehension, thus making it useful for scoring both dimensions within the rubric.

The mastery levels (codimensions) were assigned numbers ranging from one to four with designations of novice, emerging, proficient, and exemplary, respectively. Levels one to four were defined based on given criteria of what should be included in a student’s answer to qualify for a given score. The dimension *synthesis*, rather than examining questions from a unidimensional perspective, assessed students’ ability to consistently apply all dimensions of Johnstone’s triangle in their exploration of IMFs. This category considered its counterparts in a collective manner, and, in the case of the post-assessment, also considered the question pertaining to application-based skills gained during the laboratory practical examination (refer to question 6 in Supporting Information Section SIA). This rubric was better suited for the pre- and postlab assessment. Minor revisions were required for use on other assignments.

### Initial Rubric Norming and Scoring

To ensure consistency and agreement, evaluators applied the rubric to a sample of 10 assignments. The norming process involved training, discussion, sample scoring, calibration, and consensus. Training introduced the dimensions, descriptions, and scoring notation. For example, the notation A4 was used to denote evidence of exemplary level performance in the macroscopic dimension, and a C2 score meant that a student demonstrated an emerging mastery level of the microscopic dimension. Evaluators discussed their interpretations of the descriptions and made the necessary revisions to reflect the agreed common understanding. The evaluators then worked independently to score the sample assignments. Sample scoring was followed by the calibration process where evaluators compared and discussed any discrepancies and made adjustments to the rubric.

**Figure 6:**
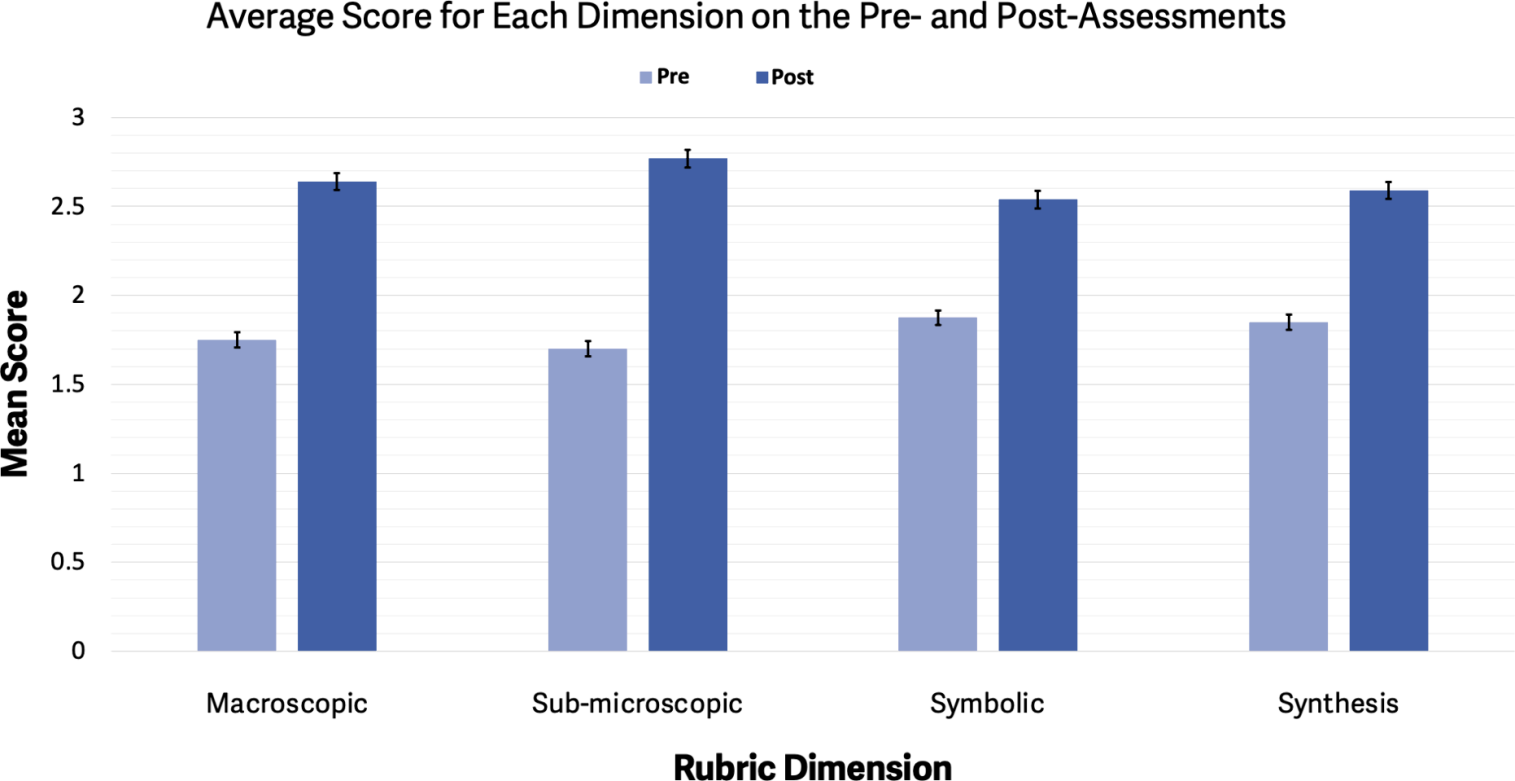
Comparison of the mean scores across dimensions on the pre- and post-assessments.

The rubric was used to assess a sample pool of pre- and post-assessments (n = 79). Each student artifact was anonymous with a numerical identifier assigned accordingly to both the pre- and post-assessments. The preassessment sample group included completed quizzes from 40 students. The post-assessment group included a single participant who had not taken the preassessment, and two students (numbers 34 and 39) participated only in the preassessment. Thus, the post-assessment sample size was 39 individuals. Scores for each question were recorded for each applicant. The rubric was then utilized to score the post-assessment, and, as shown in Figure 6, individual scores pertaining to each question were compared to those obtained from the preassessment evaluation. The students who did not complete both assignments were excluded from the comparative analysis of the pre- and post-assessments.

### Scoring Results Analysis

In this section, we demonstrate how statistical analyses may be used to gain insight into students’ progress in overcoming the IMFs threshold concept at the end of the course. Due to our smaller class sizes, we present this step as a demonstration of how one can use the rubric scores to analyze and visualize students’ progress.

Average scores for each rubric dimension were calculated by summing the codimensions values and dividing by the total number of students. Figure 6 summarizes the average score and standard error for each dimension for the two assessments. Visually, it was evident that the average scores on the post assessment were higher than in the preassessment across all the dimensions. To analyze whether the differences of the means were significant, a paired *t* test was used to compare the means of the pre- and post-assessment scores for each dimension of the ASSR. A paired *t* test statistic can be calculated as  where *d̅* denotes the mean of the differences between the paired observations, μ_*d*_ denotes the difference between the population means, *s*_*d*_ denotes the sample standard deviation, and *n* denotes the number of pairs. When the null hypothesis is μ_*d*_ = 0, the test statistic can simply be calculated as . Before using a test, it is always important to check the assumptions and how your data satisfies or approximates them. For example, when utilizing a paired *t* test, the differences between the paired observations are assumed to be normally distributed.^29^

Pre- and post-assessment scores for each dimension, alongside total scores, were compiled and analyzed in RStudio. The link to the source code and the Excel file used is provided in Section SID (Supporting Information). Normality of the differences between pairs was first assessed using the Shapiro-Wilk test of normality. While the distributions of the differences for the microscopic dimension and the total scores were approximately normal, some dimensions had better fit than others. For didactic purposes, the paired *t* test was still performed, but we discuss important limitations of this approach at the end of this section. Test statistics, associated p-values, and 95% confidence intervals were subsequently generated for pre- and post-assessment scores of each dimension.

All analyses utilized an α of 0.05 to determine significance. The null hypothesis (H_0_) was H_0_: μ_*d*_ = 0, while the alternative hypothesis (H_A_) was H_A_: μ_*d*_ ≠ 0. For the macroscopic dimension, there was a significant difference in the ASSR-assigned score between the preassessment (*x̅* = 1.76, s = 0.63) and the post-assessment (*x̅* = 2.66, s = 0.71); (t(37) = −7.22, p = 1.44 × 10^–8^, 95% CI [−1.15, −0.64]). Similarly, for the microscopic dimension, there was a significant difference in the mean score between the preassessment (*x̅* = 1.71, s = 0.69) and the post-assessment (*x̅* = 2.79, s = 0.74); (t(37) = −6.67, p = 7.77 × 10^–8^, 95% CI [−1.41, −0.75]). For the symbolic dimension, there was a significant difference in the mean score between the preassessment (*x̅* = 1.92, s = 0.78) and the post-assessment (*x̅* = 2.55, s = 0.76); (t(37) = −4.01, p = 2.8 × 10^–4^, 95% CI [−0.95, −0.31]). Finally, for the dimension of synthesis, there was a significant difference in the average score between the preassessment (*x̅* = 1.90, s = 0.56) and the post-assessment (*x̅* = 2.61, s = 0.59); (t(37) = −6.31, p = 2.38 × 10^–7^, 95% CI [−0.94, −0.48]). These findings demonstrate a way to quantify the significance of determined mean differences between the pre- and post assessments. Thus, we provide insight into learning gains associated with each dimension.

A key limitation of this analysis is the sample size of only 38 pairs. Three students were removed from the data set, as they did not complete both the pre- and post-assessments. This small sample size may be contributing to the variability in normality fit, as illustrated by the Shapiro–Wilks tests depicted in Section SIC (Supporting Information). For instructors interested in statistically assessing pedagogical materials and student performance, careful consideration should be made for the selected test based on the data’s alignment with the necessary assumptions. A nonparametric analogue of the paired *t* test, namely the Wilcoxon signed-rank test, may be an option to consider.^30^

Despite these limitations, this analysis shows that data obtained from the application of the rubric can be utilized to provide insight into meaningful progress in overcoming a threshold concept.

### Generalization of the Rubric

**Figure 7:**
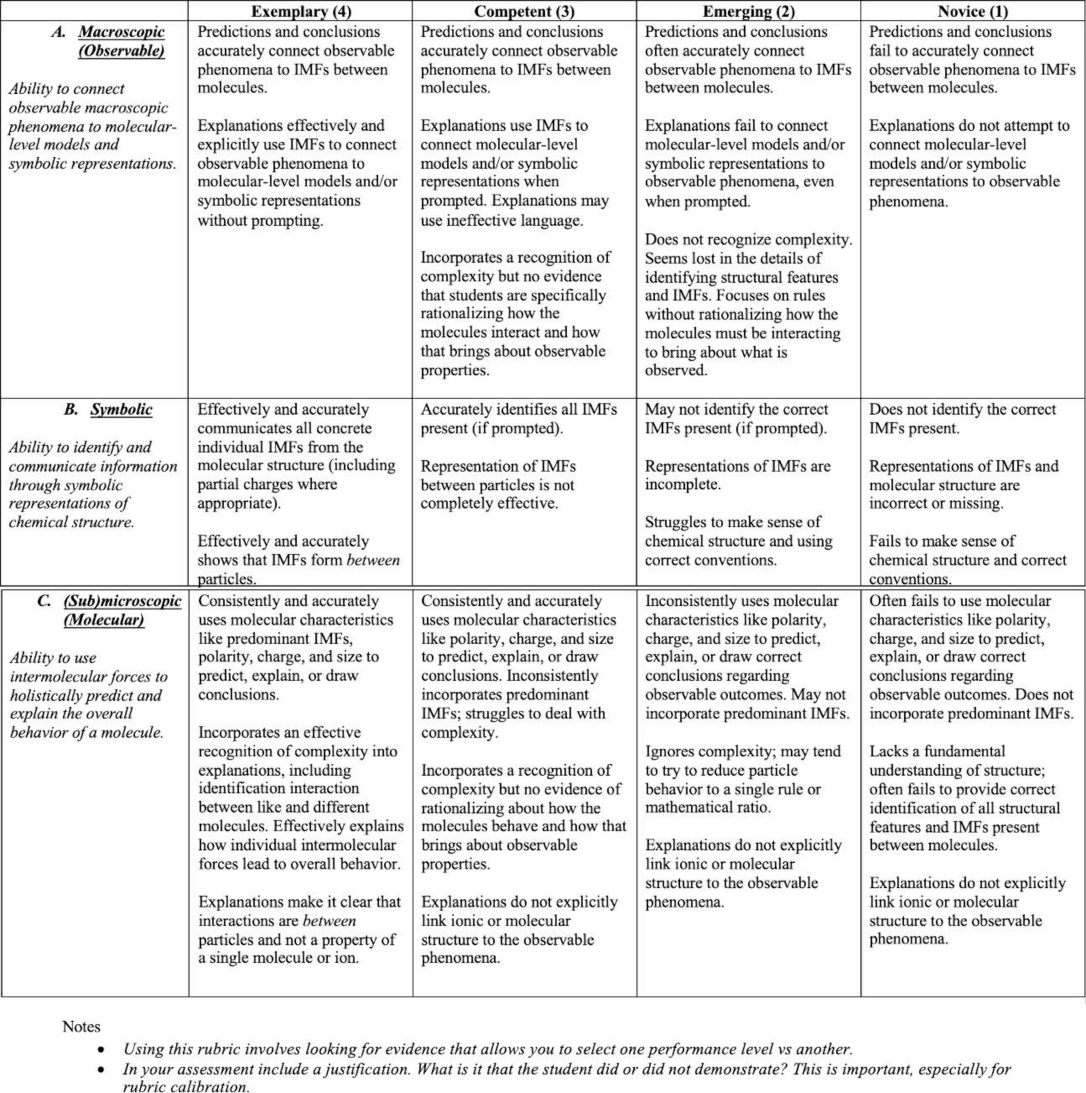
Threshold Concept Assessment Rubric designed for application across different Chem 202L assignments.

Following the norming and revision of the preand post assessment rubric, we moved to generalizing the rubric to be applicable to other assignments. The thought process for the generalization was the creation of a rubric that can be used as a guide for making ASSRs. The process of scoring samples, discussions, and calibration was repeated several times until consensus was reached. More importantly, the criteria were generalized to fit different modes of assessments. The developed Threshold Concept Assessment Rubric (TCAR), see Figure 7, is guided by the dimensions of Johnstone’s triangle, and the mastery level criteria denote expectations for student comprehension of IMFs across the different assignments. Furthermore, the TCAR eliminates the dimension of *synthesis* because the generalization of scoring criteria integrates the synthesis expectations into the three dimensions to facilitate seamless navigation around the triangle. The TCAR is considerate of not only a student’s ability to identify IMFs and relevant molecular characteristics but also the effectiveness of the language utilized to communicate such conclusions.

#### Threshold Concept Assessment Rubric (TCAR)

The generalized rubric less explicitly draws upon examples from a single assignment. Rather, it is guided by a conceptual understanding of IMFs and molecular characteristics that translates across different assignments in the course. Under this framework, assessments of student performance may be adapted per the objectives of a given assignment or course while still providing continuity between grading metrics. Though the assignment-specific rubric demonstrates that Johnstone’s triangle framework can be utilized as a focused tool for student assessment, the generalized rubric demonstrates the applicability of the framework across assignments and as an evaluative tool for impact of a pedagogical intervention.

**Figure 8:**
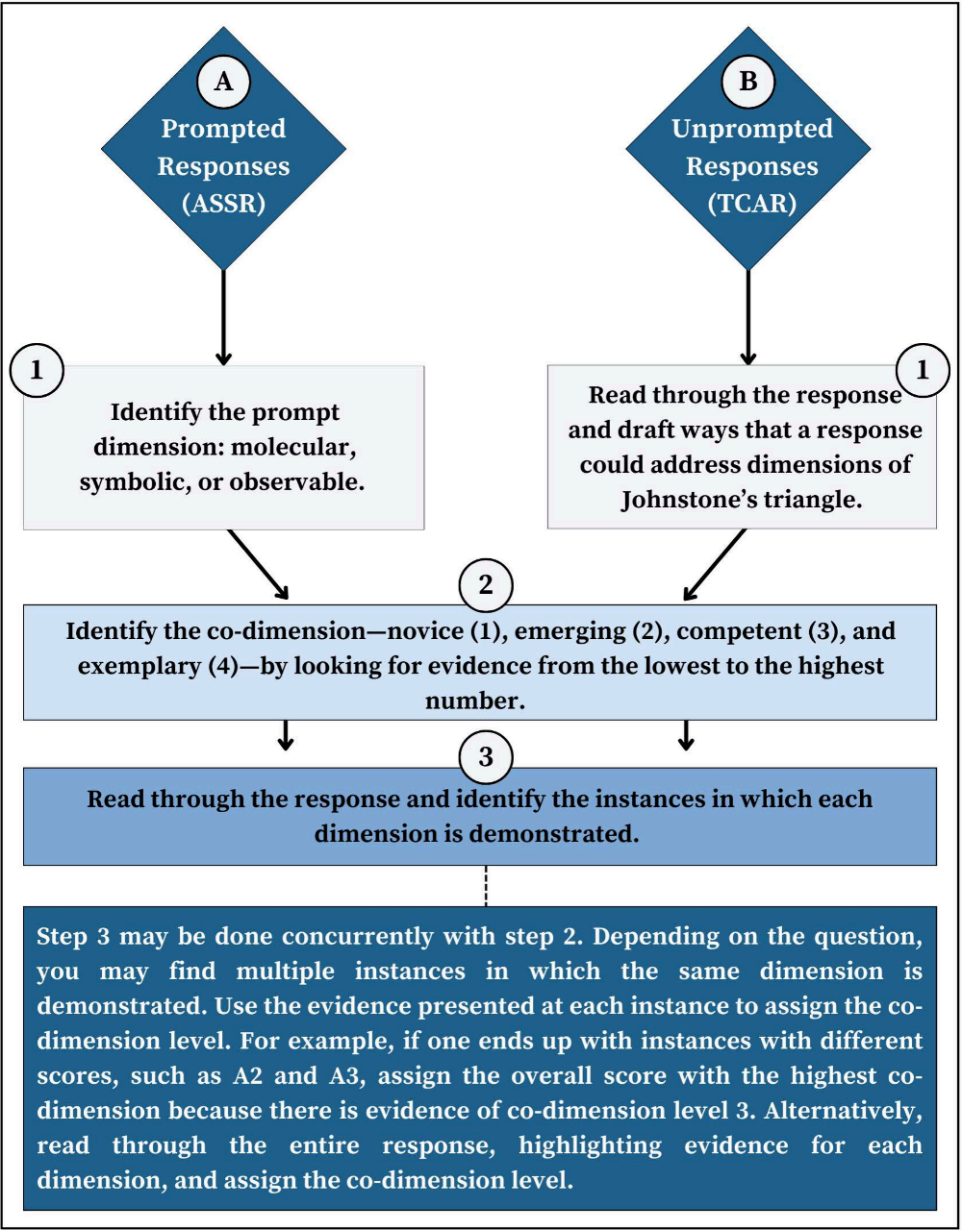
Guide to using the rubric for scoring students’ work.

The flowchart in Figure 8 provides a guide to using the TCAR to score students assignments. The chart provides two entry paths depending on whether a question is considered to include a direct prompt or not. Direct prompt questions ask demonstration of specific connections of the Johnstone’s triangle. For example, a question that asks students to represent the types of IMFs present in a molecule is considered a direct prompt. Conversely, questions that ask for explanations of observable phenomena without mentioning the use of IMFs are considered indirect, or unprompted, questions. Unprompted questions require more sifting through the response to identify the dimensions. After identifying the dimensions in step 1, the two starting paths converge. The inclusion of the two paths is designed to provide flexibility in using the flowchart with both an assignment specific rubric and a generalized rubric. Scoring examples are included in Section SIE (Supporting Information).

### Summary of the Rubric Development Process

**Figure 9:**
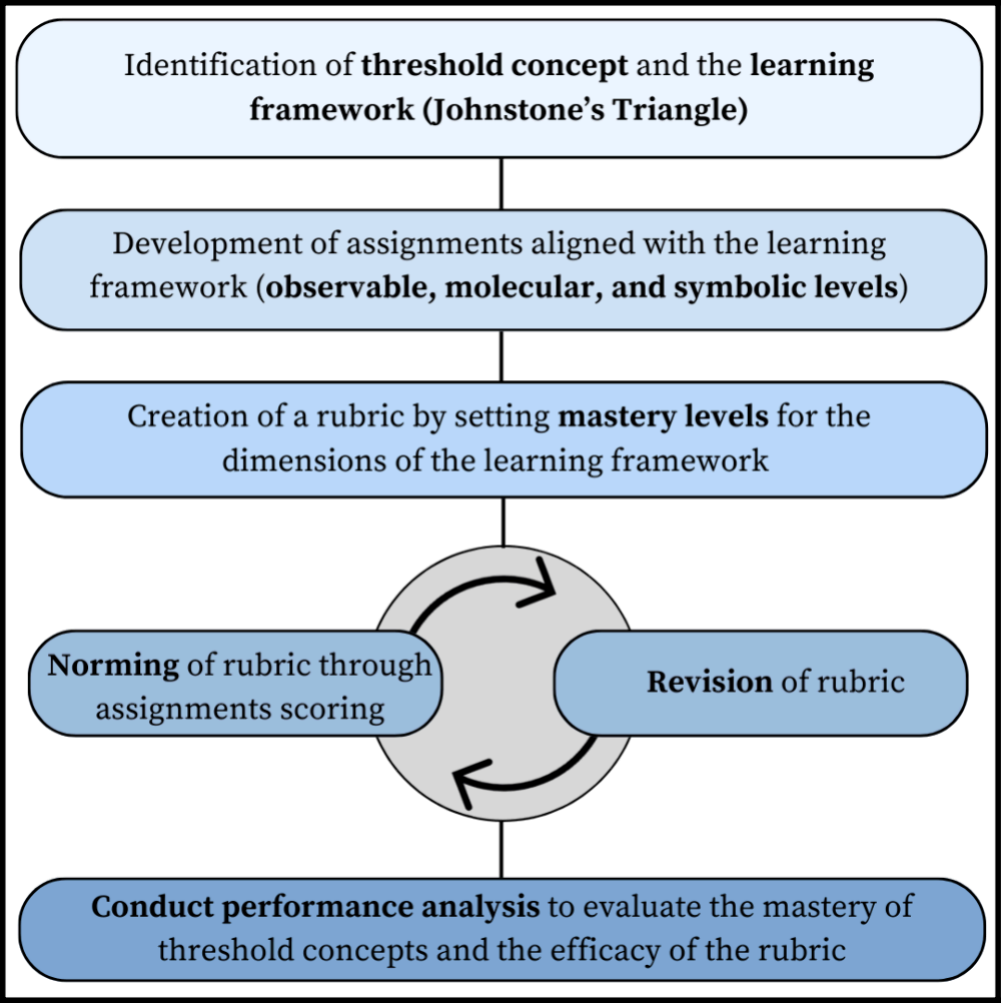
Flowchart depicting the development of a TCAR based on the Johnstone’s triangle learning framework.

A flowchart in Figure 9 summarizes the rubric development process. While the rubric was developed using the IMFs as the threshold concept, the process can be adapted to a different concept. The two main areas to adjust are the threshold concept and the learning framework for the concept.

## Conclusions

This study demonstrates that in identifying a key threshold concept, rubrics considerate of the teaching and learning framework of the course, such as the Johnstone’s triangle, can be developed and utilized to holistically assess students’ longitudinal learning growth. Application of the ASSR enabled us to quantify student progress across the dimensions of the Johnstone’s triangle. While the rubrics were developed based on an IMF threshold concept, the approach can be applied to any other concepts. The key factors are identifying a learning framework and designing assignments aligned with the framework. Threshold concepts within chemistry are a characteristic of topics across the educational spectrum.^16^ Accordingly, a repository of pedagogical tools considerate of specific impediments to student success provides educators with a basis for designing lessons, activities, and self-scoring devices. Continued improvements to the rubric design process will allow for application to more complex, multifactorial concepts commonly encountered by students in advanced chemistry courses.

In addition to providing an assessment tool, this study challenges educators to think deeply about pedagogical tools for helping students with overcoming threshold concepts. The development and application of the rubric quantified the progress toward threshold crossing among the sample population and highlighted areas in which course content could be supplemented. The TCAR development and application approach supports both instructors and students in gaining informative feedback about the learning process.

## Supporting Information Available

Sample assessment quiz (Section SIA); threshold concept assessment rubric (Section SIB); normality assessment of the paired *t* test (Section SIC); source code, scoring data, and downloadable rubrics (Section SID); rubric application examples (Section SIE) (PDF)
