# Supplementary material for: Developing a Threshold Concept Assessment Rubric: Using the Johnstone’s Triangle Framework for Understanding Intermolecular Forces
Source: J Chem Educ. 2024 Oct 2;101(11):4694–703. doi: 10.1021/acs.jchemed.4c00236 (PMC11562578; doi:10.1021/acs.jchemed.4c00236)
Supplement: Supplementary file 1 — ed4c00236_si_001.pdf [file ed4c00236_si_001.pdf]

Developing a Threshold Concept Assessment Rubric: Using the Johnstone's Triangle Framework for Understanding Intermolecular Forces

Simbarashe Nkomo* and Alia Bly

Division of Natural Science and Mathematics, Oxford College of Emory University, Oxford,Georgia 30054

E-mail: snkomo@emory.edu

Supporting Information Available

SI A. Sample Assessment Quiz

A downloadable PDF copy will be made available via the GitHub repository.

Chem 202L post assessment Name______________________

1) Draw and label diagrams to best represent the intermolecular forces:

a) like molecules of propan-2-one

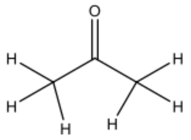

b) propane-2-one and water

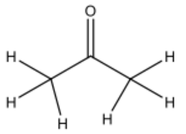

c) like molecules of fluoromethane (CH_3F)

2) Circle any of the following pairs of molecules that can H-bond with each other. Draw the H-bond where applicable.

a) 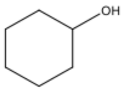 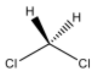

b) 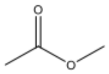 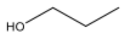

c) 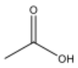 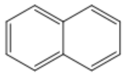

3 a) Ice floats on water. Explain why ice ($H_2O_{(s)}$) is less dense than water ($H_2O_{(l)}$)?

b) Which of the two molecules (CH_3F and HF) do you expect to have a higher boiling point?

4. Miscible liquids combine to form a homogeneous mixture, and immiscible liquids separate into different layers. Circle what you think of the miscibility of the combinations below.

a) water and ethanol CH_3CH_2OH

miscible immiscible

Justify your answer:

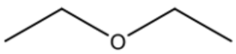

diethyl ether

b) water and diethyl ether

miscible immiscible

Justify your answer

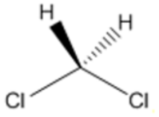

c) water and dichloromethane

miscible immiscible

Justify your answer:

5. Using drawings illustrate what happens when NaCl(s) dissolves in water.

6. Application from lab practical: You have a mixture of acetaminophen and caffeine dissolved in dichloromethane (DCM).

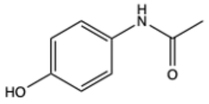

acetamenophen

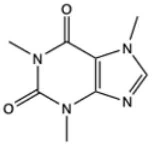

caffeine

a) How would you treat this mixture to remain with caffeine in DCM?
b) Justify your approach in part (a).

Figure S1: Sample lab quiz.

SI B. Threshold Concept Assessment Rubric (TCAR)

A downloadable PDF copy will be made available via the GitHub repository containing all study materials (see Supporting Information D for the link).

SI C. Normality Assessment of the Paired T-test

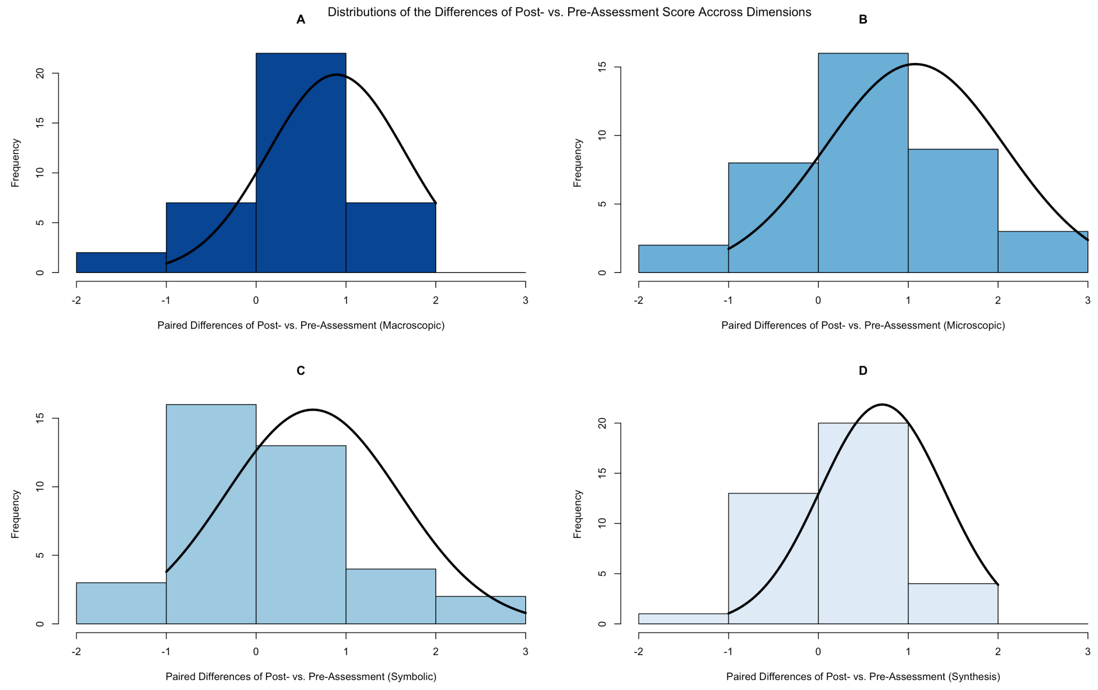

Figure S2: Examination of the normality of the paired differences between pre- and post-assessment scores in each dimension of the ASSR. In each figure, the black distribution is a scaled normal distribution, and the histogram is the sampling distribution.

Visualizations were generated in RStudio. The R source file is available in the GitHub repository provided in part D of the Supporting Information.

SI D. Source Code, Lab Quiz, and Downloadable Rubrics

For access to the source code and other materials, please visit

`https://github.com/AliaBly/ASSR_Project_Data.git`

PDF versions of both the ASSR and the TCAR are also available for download in the repository. To report access issues, please contact the corresponding author.

SI E. Examples of Using the Scoring Rubrics

Samples of the scoring process

Question 3b (Macroscopic level)

b) Which of the two molecules (CH_3F and HF) do you expect to have a higher boiling point? Explain your reasoning.

CH_3F stronger IMF, larger molecule causes greater London Dispersion forces

Fluoromethane has a molar mass of 34.03g/mol and a boiling point of -109.1°F, whereas Hydrogen fluoride has a lower molar mass of 20.10 g/mol and a higher boiling point of 67.1 °F. The correct prediction is that HF has a boiling point because it exhibits hydrogen bonding, which is a much stronger interaction when compared to the predominance of London dispersion forces attributed to the greater molar mass of fluoromethane.

We now analyze and score the student's response using the rubric. The observable phenomenon in this question is the boiling point and it is at a macroscopic level. From the rubric, we will assign the letter A for the macroscopic dimension. Next, we seek to assign the codimension level. The student fails to demonstrate the competence level by making an incorrect prediction. Taking their incorrect prediction in mind, we see that the student makes a correct connection between stronger IMFs--due to greater molar mass-- to higher boiling point. This matches codimension level 2 of emerging. The final score is A2.

Question 4c (Macroscopic level)

4. Miscible liquids combine to form a homogeneous mixture. Immiscible liquids separate into different layers (phases). Circle what you think of the miscibility of the combinations below. Explain your reasoning.

[illegible]

c) Water and dichloromethane (
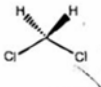

)

Miscible Immiscible

they do not have like IMFs. Cl is also more EN which contributes to higher polarity.

This question focuses on the connection between the molecular view and macroscopic level. At the macroscopic level (A), the student correctly predicts the immiscible behavior, connects with IMFs, and points outs out polarity due to the presence of the more electronegative chlorine atom. However, there is no explanation to connect the effect of like or unlike IMFs and high polarity to why the two compounds are immiscible. The response satisfies the prediction and connection to IMFs criteria for the emerging codimension (2). It fails under competent criterion because of lack of correctly connecting IMFs and high polarity to immiscibility. The response is scored A2, which corresponds to emerging level at the macroscopic dimension.

Question 1a (Symbolic level)

1.Draw and label diagrams that best represent the intermolecular forces between two molecules described below. Explain your answer for the IMF representations between molecules.

a) Like molecules of propan-2-one

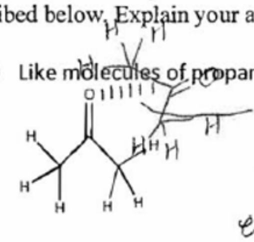

O - highly electronegative. It will drag the electrons in C=O bond towards itself, making it partially negative and carbon partially positive. Dip-dip interaction forms between these two sites.

This question prompts students to demonstrate the connection between the symbolic representation and the molecular levels of the Johnstone's triangle. Since the prompt is a symbolic representation, the student's ability to connect the levels is scored at that level (B). The response correctly identifies dipole-dipole interactions, which clears the bar for codimensions 1 and 2. The explanation is consistent with formation of partial charges leading to dipole-dipole interactions but are not represented in the symbolic illustration. It therefore falls short for codimension 4 (exemplary) and is scored at the competent level (3), which gives us an overall score of B3.

The exemplary level includes effective communication, which includes both symbolic communication and terminology or vocabulary. In the response above uses 'drag' for unequal distribution of electrons in a polar bond. Even though this communicates a correct mental model for the student, the term is not a conventional one in the subject content area. If one is using the rubric as a tool for improving the teaching process and supporting students in reaching expert level, it is important to highlight the use of effective communication consistent with conventions of the field. While others may argue that this introduces bias against those that are not first language English speakers, it is important to emphasize that using this as an assessment tool has no bearing on a student's grade. The scoring provides feedback on the faculty to model or include a discussion of terminology. However, if one decides to use the rubric as a grading rubric then the issue of bias will need to be addressed to make the rubric more inclusive. We developed the

rubric to function as a tool that informs our teaching and learning process towards supporting students in moving to expert level.

Question 5 (Molecular level)

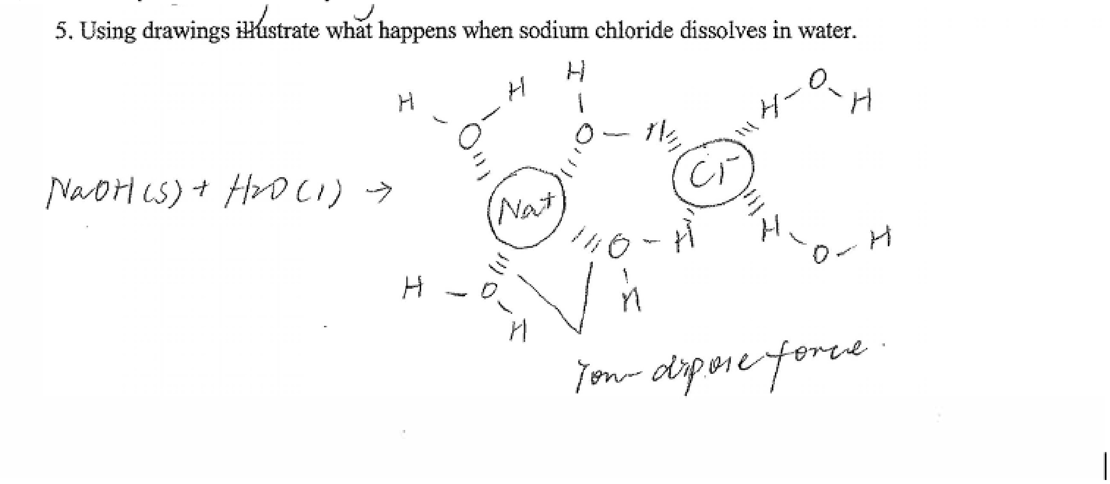

The prompt is rooted at the microscopic level because it is asking the students to illustrate their understanding of the molecular level processes. The question will, therefore, be scored at the molecular level (C). The student clearly demonstrated the existence of solvated ions and the type of interactions. Furthermore, the illustration clearly and effectively demonstrates their understanding of molecular level interactions by the orientation of water molecules around the cation and anion. While the question lacks the explanation prompt that would provide a more holistic insight into their mastery, the representations provide evidence of expert level understanding (4). The overall score is C4. During the scoring and norming processes of the rubric insightful suggestions such adding the explanation prompt may come up and provide directions for improving the questions or rubric.

Question 6 (Synthesis in ASSR)

Most of the examples used so far included direct prompts for the intended connections the students we asked to demonstrate. This example provides an example of a questions where there is no direct prompt to the different dimensions of the Johnstone's triangle. This is not an additional dimension to the triangle, but a way for assessing students' abilities to use the three dimensions of the Johnstone's triangle to effectively communicate their understanding of a physical or chemical phenomenon. As shown in Figure 3, students already have exposure to liquid-liquid extraction techniques.

6. You have a mixture of Acetaminophen and caffeine dissolved in dichloromethane (DCM).

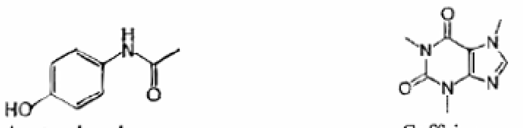

Acetaminophen Caffeine

a) How would you treat this mixture to remain with caffeine in DCM?

~~Use NaOH(aq) to the original Solution an~~

Use NaOH (aq) to extract the acetaminophen from the original Solution.

b) Justify your approach in part a).

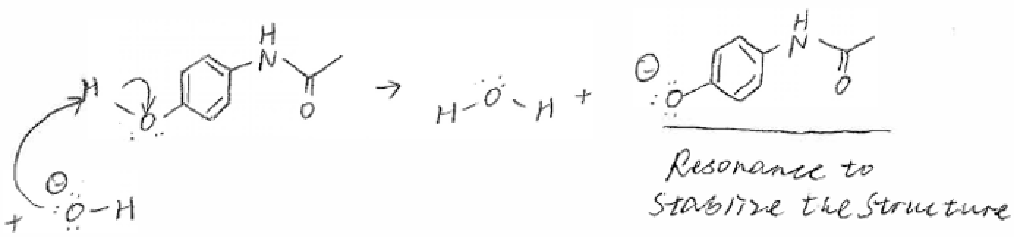

NaOH, as a Strong base, Can deprotonate the OH group in acetaminophen to make it charged. Hence, the new charged acetaminophen Can form Strong ion-dipole force with water and hence become soluble. As there's no acidic part in caffeine molecule to be deprotonated, caffeine will remain uncharged and Stay in DCM while acetaminophen can be extracted ~~using~~ from DCM using water.

The observable in this question is the extraction of the two compounds into different solvents- water (H_2O) and dichloromethane (DCM). In part (a), the student demonstrates an understanding of molecular structure by identifying presence and absence of an acidic proton in acetaminophen and caffeine, respectively. The equation in part b, illustrates molecular transformations using symbolic representations. The explanation connects the molecular and symbolic levels to the

observable phenomenon. The overall score is D4, which is an exemplary level demonstration of the student’s ability to use the Johnstone’s framework.
